# Supplementary material for: A Matter of Shape: Contact Area Optimization in Soft Lubricated Impact
Source: Tribol Lett. 2026 Jan 23;74(1):15. doi: 10.1007/s11249-026-02108-1 (PMC12830445; doi:10.1007/s11249-026-02108-1)
Supplement: Supplementary file 1 — (pdf 505 KB) [file 11249_2026_2108_MOESM1_ESM.pdf]

A Matter of Shape: Contact Area Optimization in Soft Lubrication
Supplementary Material

Joaquin Garcia-Suarez[1]

Institute of Civil Engineering, Institute of Materials, École Polytechnique Fédérale de Lausanne (EPFL), CH-1015 Lausanne, Switzerland

---

Contents

1 Methods **1**

1.1 Dry contact mechanics 1

1.2 Fluid-mediated approach of indenter to smooth rigid substrate . 4

1.3 Non-dimensionalization and finite-difference discretization 7

2 Detailed derivations and extra results **9**

2.1 Pressure distribution and comparison to contact mechanics . . . 9

2.2 Comparing bubble sizes 10

1. Methods

1.1. Dry contact mechanics

Consider an elastic halfspace indented by a rigid object. The impact indentation problem is equivalent to the static one when the total force being applied on the indenter is replaced by its inertia, which must be absorbed by the strain energy associated to the deformation of the body being indented. This setting assumes that the characteristic time of wave propagation in the solid is much shorter than the characteristic time of the viscous approach, so transient

[1]Correspondence: joaquin.garciasuarez@epfl.ch

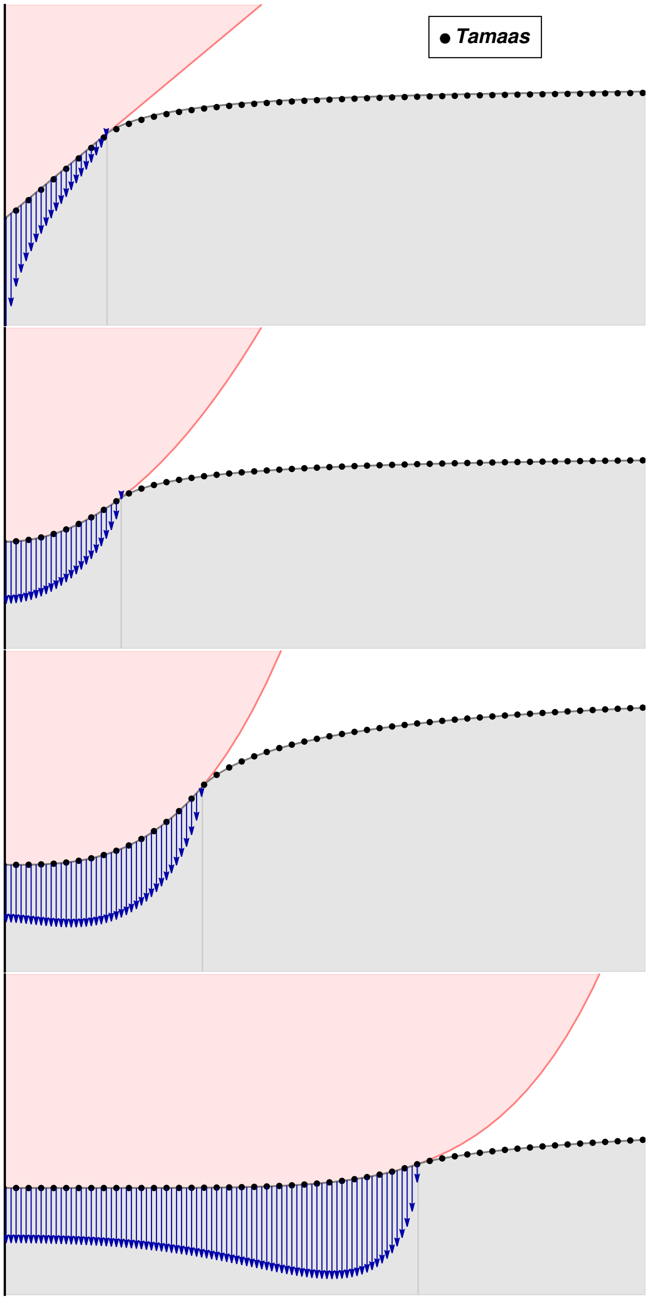

Figure S1: From top to bottom: dry indentation setting for linear, parabolic, cubic and hexic tip shapes. The pressure profiles shown are obtained from Tamaas simulations [1]. The deformed halfspace surface is represented by the analytical solution [2] with superimposed dots being the numerical Tamaas results. The darker vertical mark represents a, the contact radius.

inertial effects in the solid can be neglected. The compendium by Popov and colleagues [3] includes the general solution for an indenter whose profile is given by a monomial $\sim r^n$, in an axisymmetric setting. They also notice that the pressure distribution developing under the indenter features the maximum at the center ($r = 0$) if $n \leq 2$, while the peak pressure moves the closer to the contact edge the higher the exponent $n > 2$.

Consider an indenter of mass m impacting the rigid surface. The deformation $\delta(t)$ (indentation depth) during contact is related to the elastic force $F(t)$, given by contact mechanics theory [3]:

$$F(t) = E^* \frac{2n}{n+1} \left[\frac{\kappa(n)}{2k} \right]^{1-n} \delta(t)^{n+1} \,, \tag{1}$$

where:

- E^* is the effective Young's modulus, defined as $E^* = \frac{E}{1-\nu^2}$, with E being the Young's modulus and ν the Poisson's ratio of the material.
- The function $\kappa(n)$ is referred to as the "stretch factor" [3], it depends on the gamma function, Γ:

$$\kappa(n) = \sqrt{\pi} \frac{\Gamma(n/2+1)}{\Gamma(n/2+1/2)} \,. \tag{2}$$

- k is the geometric constant of the indenter, such that the profile is written as $f(r) = r^n/2k$, the units being a function of the exponent.
- $\delta(t)$ is always positive during contact.

Note that the contact stiffness, for a fixed level of indentation δ, increases with greater n, meaning that the blunter the indenter and the more spread out the contact, the more difficult to indent.

Assuming that the wave propagation over the full object happens much faster than the characteristic time associated to the build-up of lubrication pressures in the thin film [4], applying Newton's second law in the vertical direction to

the center of mass of the indenter yields:

$$m\ddot{\delta}(t) = -F(t)\,, \tag{3}$$

from this balance, one can also assess the energy conversion from kinetic to elastic energy. The dry-contact theory delivers the value of the maximum depth:

$$\delta_{\max} = \left(\frac{[\kappa(n)/2k]^{1/n}(2n+1)(n+1)mV_0^2}{4n^2 E^*} \right)^{\frac{n}{2n+1}} . \tag{4}$$

The corresponding maximum contact radius is

$$a_{\max} = (2\delta_{max}k)^{1/n} . \tag{5}$$

It is easy to recover the Hertzian (parabolic indenter) case [5] fixing $k = R$ and $n = 2$.

The exact solution of the mixed-boundary value problem [6] yields the fields of interest (surface pressure, surface displacement...) in terms of integrals that can be evaluated for the different values of n. To obtain the results for different geometries in the dry case, we follow Sneddon [2]: his version of the integrals are evaluated symbolically in Mathematica [7]. For further reassurance, numerical solutions are also obtained using Tamaas [1], a boundary element code optimized for contact mechanics problems (see Figure S1).

1.2. Fluid-mediated approach of indenter to smooth rigid substrate

Analyzing this phenomenon requires suitable descriptions of the indenter deformation (elasticity) and the viscous response of the air (lubrication). This work is restricted to the quasi-static case, meaning that the characteristic time of the viscous phase of the approach is much longer than the characteristic time associated to wave propagation over the indenter tip [4]. This allows to borrow the numerical implementation by V. Bertin [5] of the implicit solver proposed in Ref. [8], featuring an efficient discretization scheme of the solid-fluid interaction problem based on the fundamental solution proposed by David et al. [9] and a finite-difference discretization for Reynolds lubrication equation, elegantly implemented in a concise Python code. The slightly modified version used here only adds the

possibility of changing the indenter shape; the original code can be accessed at `https://github.com/vincent-bertin/elastohydrodynamic-bouncing`.

The lubrication equation is:

$$r\frac{\partial h(r,t)}{\partial t} = \frac{1}{12\eta}\frac{\partial}{\partial r}\left(rh^3(r,t)\frac{\partial p(r,t)}{\partial r}\right) , \tag{6}$$

where:

- $h(r,t)$ is the film thickness as a function of the radial coordinate r and time t.
- η is the viscosity of the fluid.
- $p(r,t)$ is the pressure field within the thin film.

The relative height depends on the shape of the leading edge profile and the deformation induced by the lubrication pressures. The profile at any given time is expressed as

$$h(r,t) = D(t) + \frac{r^n}{2k} - w(r,t) , \tag{7}$$

where $D(t)$ represents the vertical position of the center point and w is the elastic vertical deformation, which is computed using a kernel [9]:

$$\begin{aligned} w(r,t) &= -\frac{4}{\pi E^*}\int_0^\infty \mathcal{M}(x,r)p(x,t)dx \\ &= -\frac{4}{\pi E^*}\int_0^\infty \frac{x}{x+r}K\left(\frac{4xr}{(x+r)^2}\right)p(x,t)dx , \end{aligned} \tag{8}$$

where p is the pressure distribution acting over the leading edge of the indenter and K is the complete elliptic integral of the first kind.

Following Ref. [5], We choose the characteristic values of the problem to be those furnished by dry contact mechanics. Their explicit form in terms of the physical parameters depends on the exponent n. Assuming that the undeformed

profile shape is given as $f(r) = r^n/2k$, they have the form below in all cases.

Characteristic vertical length [3]: $$\mathcal{H} = \left(\left(\frac{\kappa(n)}{2k} \right)^{1/n} \frac{(2n+1)(n+1)}{4n^2} \frac{mV_0^2}{E^*} \right)^{\frac{n}{2n+1}} \tag{9a}$$

Characteristic radial length: $$\mathcal{L} = (k\mathcal{H})^{1/n} \tag{9b}$$

Characteristic pressure: $$\mathcal{P} = \frac{2E^*}{\pi} \frac{\mathcal{H}}{\mathcal{L}} \tag{9c}$$

Characteristic time: $$\tau = \frac{mV_0}{E^* \mathcal{L} \mathcal{H}} \tag{9d}$$

A dimensionless group also springs from the equations:

Stokes number: $$\mathrm{St} = \frac{\tau \mathcal{P} \mathcal{H}^2}{12\eta \mathcal{L}^2} \tag{10}$$

These scales are selected purposefully to minimize the number changes needed to generalize the solver implementation that V. Bertin used to analyze parabolic indenters [5]. They guarantee that both sides of each dimensionless equation are $\mathcal{O}(1)$, except for the lubrication equation, eq. (6), where the Stokes number appear in its right-hand member.

Let us quickly comment on the meaning of some of the scales and on the significance of the Stokes number. $\mathcal{P}$ represents the elastic stress associated to a characteristic strain, provided that $\mathcal{H}$ and $\mathcal{L}$ characterize the vertical and radial deformation respectively. τ is the characteristic time associated to the elastic forces decelerating the approaching object. Finally, the Stokes number, St, represents the ratio between elastic and viscous pressures over the timescale of the approach, i.e., the timescale associated to the complete deceleration of the object prior to rebound. Since the elastic pressures are linked to the complete deceleration, the latter sets the scale for both geometrical length scales and time scale. This means that St$\gg 1$ given the rest of parameter values. In other words, the global indenter inertia would define the overall elastic deformation, a pre-assumption of the dry impact/contact setting (Section 1.1). This is, however, misleading. Over shorter timescales, necessarily St must be $\mathcal{O}(1)$, meaning that the viscous pressures must be ultimately the ones in charge of *locally* arresting

and deforming the indenter, even though the *global* approach-contact-rebound process happens over a much longer timescale. This is how these two facts are reconciled in practical terms: the simulations are run on the global timescale, starting at a height $\mathcal{H}$ from the interface, imparting an initial velocity $V(t=0)$, running until the velocity of the object changes sign (approach ends, rebound starts); simultaneously the timestep is chosen small enough so as to resolve the timescale over which the viscous forces in the fluid locally balance the leading edge inertia.

1.3. Non-dimensionalization and finite-difference discretization

The governing equations can be rendered dimensionless resorting to the characteristic values of the dry case Section 1.1. The dimensionless variables, using the scales in (9) become:

$$\tilde{r} = \frac{r}{\mathcal{L}}\,, \quad \tilde{w} = \frac{w}{\mathcal{H}}\,, \quad \tilde{h} = \frac{h}{\mathcal{H}}\,, \quad \tilde{D} = \frac{D}{\mathcal{H}}\,, \quad \tilde{t} = \frac{t}{\mathcal{H}/V_0}\,, \quad \tilde{p} = \frac{p}{\mathcal{P}}\,, \quad \tilde{V} = V/V_0\,. \tag{11}$$

The new dimensionless eq. (6), eq. (7), eq. (3) and eq. (8) read as follows:

$$\tilde{r}\frac{\partial \tilde{h}(\tilde{r},\tilde{t})}{\partial \tilde{t}} = \mathrm{St}\frac{\partial}{\partial \tilde{r}}\left(\tilde{r}\tilde{h}^3(\tilde{r},\tilde{t})\frac{\partial \tilde{p}(\tilde{r},\tilde{t})}{\partial \tilde{r}}\right)\,, \tag{12}$$

$$\tilde{h}(\tilde{r},\tilde{t}) = \tilde{D}(\tilde{t}) + \frac{\tilde{r}^n}{2} - \tilde{w}(\tilde{r},\tilde{t})\,, \tag{13}$$

$$\frac{d\tilde{V}(\tilde{r},\tilde{t})}{d\tilde{t}} = 4\int_0^\infty \tilde{p}(\tilde{r},\tilde{t})\tilde{r}d\tilde{r}\,, \tag{14}$$

$$\tilde{w}(\tilde{r},\tilde{t}) = -\frac{8}{\pi^2}\int_0^\infty \tilde{\mathcal{M}}(\tilde{r},\tilde{x})\tilde{p}(\tilde{x},\tilde{t})\,d\tilde{x}\,. \tag{15}$$

The discretization scheme applies a finite-difference method to solve the dimensionless equations.

1. Spatial and Temporal Discretization

A uniform spatial grid is defined as:

$$\tilde{r}_i = i\Delta\tilde{r}, \quad \text{for } i \in [0, N-1]$$

where $\Delta\tilde{r}$ is the grid size and N is the total number of radial points.

The temporal axis is discretized using a constant time step:

$$\tilde{t}^m = M\Delta\tilde{t}$$

where Δt is sufficiently small (e.g., 10^{-3} or less) for numerical stability (resolving lubrication timescale).

2. Discretized Variables

Pressure, film thickness, and deformation are discretized as:

$$\tilde{p}(\tilde{r}, \tilde{t}) = \tilde{p}_i^m, \quad \tilde{h}(\tilde{r}, \tilde{t}) = \tilde{h}_i^m, \quad \tilde{w}(\tilde{r}, \tilde{t}) = \tilde{w}_i^m$$

Velocity and sphere position are given by $\tilde{V}^m$ and $\tilde{D}^m$.

3. Discretized Thin-Film Equation

The thin-film equation (A4a) becomes:

$$\tilde{V}^{m+1} - \frac{\tilde{w}_i^{m+1} + \tilde{w}_i^m}{\Delta t} = \mathrm{St}\left[\frac{(\tilde{h}_i^m)^3}{\tilde{r}_i}\frac{\partial\tilde{p}}{\partial\tilde{r}}\bigg|_i^{m+1} + 3(\tilde{h}_i^m)^2\left(\frac{n}{2}\tilde{r}_i^{n-1} - \frac{\partial\tilde{w}}{\partial\tilde{r}}\bigg|_i^m\right)\frac{\partial\tilde{p}}{\partial\tilde{r}}\bigg|_i^{m+1} + (\tilde{h}_i^m)^3\frac{\partial^2\tilde{p}}{\partial\tilde{r}^2}\bigg|_i^{m+1}\right] \tag{16}$$

with film thickness:

$$\tilde{h}_i^m = \tilde{D}^m + \frac{\tilde{r}_i^n}{2} - \tilde{w}_i^m$$

4. Spatial Derivative Approximations

First-order and second-order derivatives are approximated as:

$$\frac{\partial\tilde{w}}{\partial\tilde{r}}\bigg|_i^m = \frac{\tilde{w}_{i+1}^m - \tilde{w}_i^m}{\Delta\tilde{r}},$$

$$\frac{\partial\tilde{p}}{\partial\tilde{r}}\bigg|_i^{m+1} = \frac{\tilde{p}_{i+1}^{m+1} - \tilde{p}_i^{m+1}}{\Delta\tilde{r}}$$

$$\frac{\partial^2\tilde{p}}{\partial\tilde{r}^2}\bigg|_i^{m+1} = \frac{\tilde{p}_{i+1}^{m+1} - 2\tilde{p}_i^{m+1} + \tilde{p}_{i-1}^{m+1}}{\Delta\tilde{r}^2}$$

5. Boundary Conditions

Symmetry at $\tilde{r} = 0$:

$$\left.\frac{\partial \tilde{p}}{\partial \tilde{r}}\right|_{\tilde{r}=0} = 0 \implies \tilde{p}_1^n - \tilde{p}_0^n = 0$$

Decay at large radius:

$$\tilde{p}_{N-1}^n = 0$$

6. Integral Equation of Elasticity

Elastic deformation $\tilde{w}_i^m$ is computed using:

$$\tilde{w}_i^m = -\frac{8}{\pi^2}\left[\tilde{p}_0^m \int_0^{\Delta r/2} \frac{x}{x+\tilde{r}_i} K\left(\frac{4x\tilde{r}_i}{(x+\tilde{r}_i)^2}\right) dx \right.$$
$$\left. + \sum_{j=1}^{N-1} \tilde{p}_j^m \int_{\tilde{r}_j-\Delta r/2}^{\tilde{r}_j+\Delta r/2} \frac{x}{x+\tilde{r}_i} K\left(\frac{4x\tilde{r}_i}{(x+\tilde{r}_i)^2}\right) dx \right]$$

7. Newton's Second Law for the Indenter

Velocity and position updates:

$$\frac{\tilde{V}^{m+1} - \tilde{V}^m}{\Delta t} = 4 \sum_{i=0}^{N-1} \tilde{p}_i^{m+1} \tilde{r}_i \Delta \tilde{r}$$

$$\frac{\tilde{D}^{m+1} - \tilde{D}^m}{\Delta t} = \tilde{V}^{m+1}$$

The recasting of the equations into a linear system of equations (the unknown vector containing the values of surface deformation in the solid and the pressure in the fluid) that can be solved with standard numerical methods follows the scheme presented in [8].

2. Detailed derivations and extra results

2.1. Pressure distribution and comparison to contact mechanics

The time presented in the results is re-scaled so that $t = 0$ corresponds to the moment in which the tip of the indenter would contact the rigid substrate in the absence of intervening fluid. All the simulations herein and in the letter

correspond to St $= 1000 \gg 1$, $\Delta\tilde{r} = 0.003$ and $N = 2000$ (i.e., simulation window ranges from $\tilde{r} = 0$ to $\tilde{r} = 6$, where the boundary condition enforced), and $\Delta\tilde{t} = 0.001$ and $M_{\max} = 10000$ (in practice this number of timesteps is never reached, simulations usually stop around $\tilde{t} \approx 2$ ($M \approx 2000$) because velocity changes sign and the rebound onset is detected).

The indentation depth is taken directly from the numerical records of: we use the impactor deformation at each time t, δ_{num}, taken the value at the center, i.e., $\delta_{\text{num}} = w(0,t)$. For instance: in the parabolic case $f(r) = r^2/2R$, the pressure distribution using the analogy is $p(r,t) = 2E^*/\pi(R\delta_{\text{num}}(t) - r^2)^{1/2}$.

We also tried to predict using contact mechanics without any recourse to numerical results. To do so, we used a "constant-rate kinematic indentation" proportional to the approach velocity, δ_{kin}: for $t < 0 \rightarrow \delta_{\text{kin}} = 0$, for $t > 0 \rightarrow \delta_{\text{kin}} = V_0 t > 0$ increasing at constant rate. To be specific, using the parabolic case again, the "kinematic" indentation was computed as $p(r,t) = 2E^*/\pi(RVt - r^2)^{1/2}$. Results are presented at Figure S2, they correspond to maximum pressure (no matter where the peak happens) in all cases but $n = 1$, because the pressure is singular; the finite pressure at $r = 0.04\mathcal{L}$ is monitored instead. There is a good match for short times, meaning that at first the indentation that is experienced is predictable, the effects of deceleration are not apparent.

For longer times, the predictions worsens, particularly for higher $n > 2$. In all cases the kinematic approximation overpredicts the final pressure.

2.2. Comparing bubble sizes

Clearly, under these assumptions of localized viscous effects (St $\gg 1$) and low approach velocity, using a conic tip appears the easiest way to prevent air entrapment, based on results presented in Figure 2 in the letter. It remains to be answered how the physical size of the bubble changes for different tip geometries, assuming that physical parameters are equivalent among indenters, e.g., made of the same material (same mass and elastic properties) and somehow equivalent geometry, i.e., same maximal cross-section. For this purpose, we propose the following scenario: consider the parabolic, cubic and hexic leading edge, and

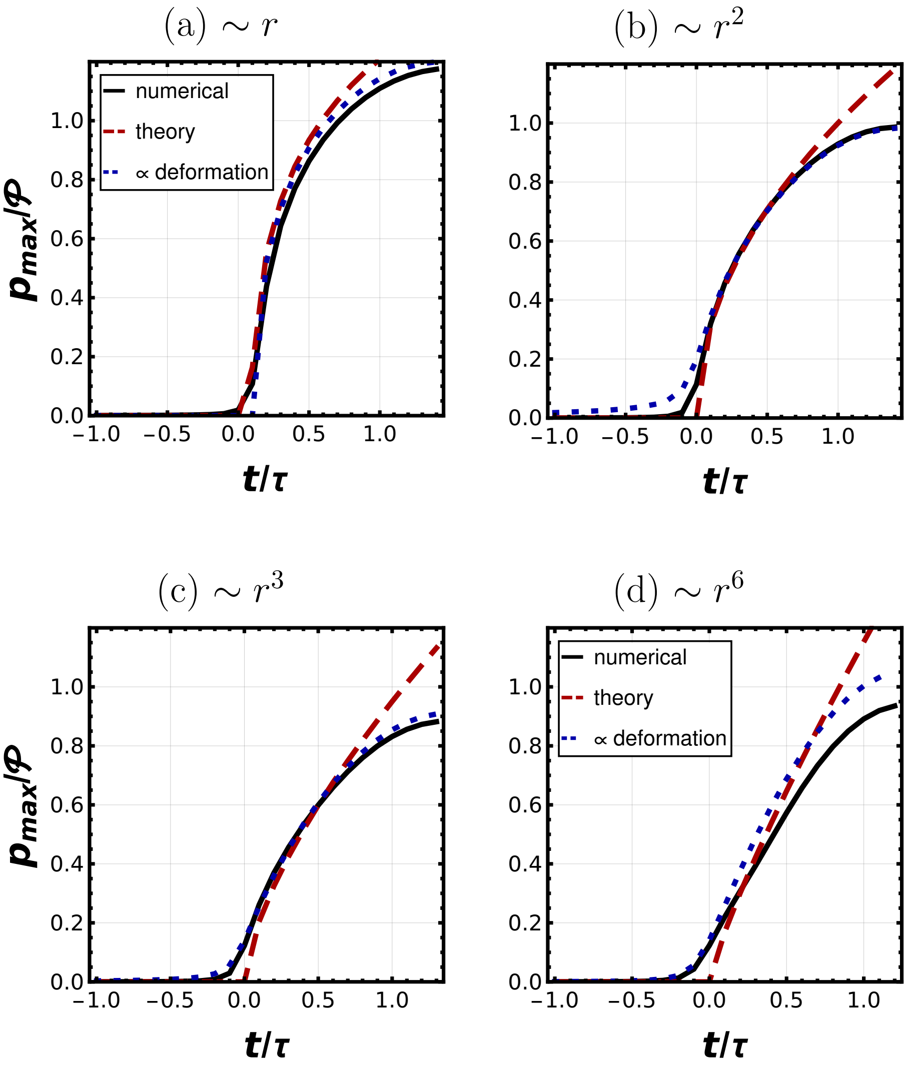

Figure S2: Maximum pressure over time according to numerical simulations, analogy using deformation numerical results and analogy using kinematic approximation to deformation. (a) Linear profile. (b) Parabolic. (c) Cubic. (d) Hexic.

assume that all three profiles match at a certain point whose coordinates are $r = R/\sqrt{5}$ and $z = h(r) = R/10$ (see Figure 3 in the letter). By doing this, we can establish an equivalence that defines a consistent value for the k in $f(r) = r^n/2k$. It follows that for $n = 2 \rightarrow k = R$, for $n = 3 \rightarrow k = R^2/\sqrt{5}$ and for $n = 6 \rightarrow k = R^5/25$. From the numerical results we have obtained the dimensionless volume of each bubble $\tilde{\Omega}_b$, computed as

$$\tilde{\Omega}_b = \pi \int_{\tilde{h}(\tilde{r},\tilde{t}_{\text{end}})} \tilde{r}^2 d\tilde{z}\,, \tag{17}$$

where, recall, $\tilde{r} = r/\mathcal{L}$, $\tilde{z} = z/\mathcal{H}$, and $\tilde{h}(\tilde{r}, \tilde{t}_{\text{end}})$ is the leading edge profile at the end of the simulation , slightly offset so that $\min\left[\tilde{h}(\tilde{r}, \tilde{t}_{\text{end}})\right] = 0$ to avoid the tiny gap that remains between the solid lowest point and $\tilde{z} = 0$ at the end of the simulation. Thus, the physical bubble size can be computed as $\Omega_b = \tilde{\Omega}_b(\mathcal{H}\mathcal{L}^2)$. As we are not interested in bubble size *per se*, but on relative sizes as the shape changes (different n), let us display the relative size of the bubble in the cubic and the hexic case with respect to the one in the parabolic case. These ratios happen to depend on the dimensionless group $\Pi = mV^2/E^*R^3$; assuming that $m/\rho R^3 = \mathcal{O}(1)$ in all geometries, it follows that $\Pi \sim (V/c)^2$, where c is any wave velocity in the solid. Then $\Pi \ll 1$ in all practical cases, which will come in handy in interpreting the results; for illustration purposes we ignore prefactors and substitute $c/V = 100$ (soft solid) and $c/V = 1000$ (stiff solid). Comparing parabolic and cubic indenters:

$$\frac{\tilde{\Omega}_b|_{n=3}}{\tilde{\Omega}_b|_{n=2}} \cdot \frac{[\mathcal{H}\mathcal{L}^2]_{n=3}}{[\mathcal{H}\mathcal{L}^2]_{n=2}} = \frac{0.019}{0.012} \cdot \frac{0.648}{\Pi^{3/35}} = \frac{1.05}{\Pi^{3/35}} \sim \left(\frac{c}{V}\right)^{3/65} = \begin{cases} 2.20, \text{ soft} \\ 3.27, \text{ stiff} \end{cases} \tag{18}$$

While for the hexic one:

$$\frac{\tilde{\Omega}_b|_{n=6}}{\tilde{\Omega}_b|_{n=2}} \cdot \frac{[\mathcal{H}\mathcal{L}^2]_{n=6}}{[\mathcal{H}\mathcal{L}^2]_{n=2}} = \frac{0.046}{0.012} \cdot \frac{0.4}{\Pi^{12/65}} = \frac{1.54}{\Pi^{12/65}} \sim \left(\frac{c}{V}\right)^{24/65} = \begin{cases} 5.48, \text{ soft} \\ 12.81, \text{ stiff} \end{cases} \tag{19}$$

The prior results indicate that the blunter the indenter, the more air is entrapped. It seems therefore that the blunter leading edge leads to a greater

extent of the region where significant viscous pressures develop, and this in turn redounds to a greater deformation and a larger bubble. The relative bubble size also increases as the material softens (lower wave velocity for the same approach velocity). Hence, there seems to be a consistent trend: to minimize the amount of entrapped air and maximize the contact area at the tip (assuming the low velocity setting and large Stokes number), it is better to use "sharper" profiles (avoid a blunt leading edge) and stiffer materials.

References

[1] L. Frérot, G. Anciaux, V. Rey, S. Pham-Ba, J.-F. Molinari, Tamaas: a library for elastic-plastic contact of periodic rough surfaces, Journal of Open Source Software 5 (51) (2020) 2121.

[2] I. N. Sneddon, The relation between load and penetration in the axisymmetric boussinesq problem for a punch of arbitrary profile, International Journal of Engineering Science 3 (1) (1965) 47–57.

[3] V. L. Popov, M. Heß, E. Willert, Handbook of contact mechanics: exact solutions of axisymmetric contact problems, Springer Nature, 2019.

[4] J. Bilotto, J. M. Kolinski, B. Lecampion, J.-F. Molinari, G. Subhash, J. Garcia-Suarez, Fluid-mediated impact of soft solids, Journal of Fluid Mechanics 997 (2024) A35. doi:10.1017/jfm.2024.820.

[5] V. Bertin, Similarity solutions in elastohydrodynamic bouncing, Journal of Fluid Mechanics 986 (2024) A13.

[6] J. R. Barber, Contact mechanics, Vol. 20, Springer, 2018.

[7] S. Wolfram, The mathematica book, Vol. 4, Cambridge University Press Cambridge, 2000.

[8] Z. Liu, H. Dong, A. Jagota, C.-Y. Hui, Lubricated soft normal elastic contact of a sphere: a new numerical method and experiment, Soft Matter 18 (6) (2022) 1219–1227.

[9] R. H. Davis, J.-M. Serayssol, E. J. Hinch, The elastohydrodynamic collision of two spheres, Journal of Fluid Mechanics 163 (1986) 479–497.
